# From Immediate Impact to Enduring Change: A Transcriptomic Comparison of tDCS’s Temporal Effects and Its Long-Term Equivalence with TMS

**DOI:** 10.3390/ijms26178634

**Published:** 2025-09-04

**Authors:** Bhanumita Agrawal, Yonatan Feuermann, Julia Panov, Hanoch Kaphzan

**Affiliations:** 1 Sagol Department of Neurobiology, University of Haifa, Haifa 3103301, Israel; bhanumita.agr@gmail.com (B.A.); yfeuermann@univ.haifa.ac.il (Y.F.); jpanov@univ.haifa.ac.il (J.P.); 2 Tauber Bioinformatics Research Center, University of Haifa, Haifa 3103301, Israel

**Keywords:** tDCS, TMS, neurostimulation, mitochondria, transcriptomics, nanopore sequencing

## Abstract

Transcranial Direct Current Stimulation (tDCS) and Transcranial Magnetic Stimulation (TMS) are neuromodulatory techniques with therapeutic potential for similar disorders; however, their molecular effects require further elucidation, and whether both strategies work in similar biological pathways is unknown. Thus, determining whether these effects are unique or shared across techniques is essential for optimizing their therapeutic applications. We investigated the long-term effects of tDCS by generating a novel transcriptomic dataset and comparing it to immediate tDCS effects and long-term TMS effects using publicly available data. Transcriptomics data were generated using nanopore sequencing on parietal cortices below the stimulation electrode of C57BL/6 mice that underwent repetitive anodal tDCS (200 µA) for 20 min over 5 consecutive days. Bioinformatics analyses were conducted on this dataset in conjunction with publicly available datasets on immediate tDCS and long-term TMS effects. Repetitive tDCS induces long-term alterations in protein translation, mitochondrial function, and cellular respiration, while TMS primarily affects calcium-mediated signaling, suggesting distinct neuromodulatory and molecular mechanisms. These findings demonstrate that tDCS and TMS elicit lasting but distinct molecular changes, highlighting technique-specific neuromodulatory effects relevant to their therapeutic applications.

## 1. Introduction

Neuromodulation techniques have emerged as transformative tools in neuroscience and medicine, offering innovative approaches for modulating brain activity and treating neuropsychiatric disorders. Among the various methods, Transcranial Direct Current Stimulation (tDCS) and Transcranial Magnetic Stimulation (TMS) have attracted significant clinical attention because of their non-invasive nature, diverse applications, and minimal side effects [1]. While both techniques lead to alterations in neuronal excitability, their underlying molecular mechanisms are still poorly understood, and despite the evidence for their beneficial effects in similar disorders, it is not yet known whether they act through similar or distinct molecular pathways.

tDCS works by generating a weak electrical current (1–2 mA) between two electrodes, anode and cathode, ultimately altering the excitability of neurons in the cortical area of stimulation [2,3]. Key factors, such as polarity, stimulation duration, current density, and the targeted brain region, significantly influence the effects of tDCS [4,5]. In principle, tDCS slightly modulates the resting membrane potential of the neurons, especially in distal compartments of dendrites and axons, without directly triggering action potential [2,6,7]. Anodal tDCS is known to depolarize the soma and axons of cortical pyramidal neurons, usually increasing cortical excitability, while cathodal tDCS is known to hyperpolarize the soma and axons of these neurons, leading to a decrease in cortical excitability [8,9]. tDCS has been shown to influence neuronal plasticity and memory consolidation and affect LTP generation [10,11,12,13]. Research also suggests that tDCS modulates energy metabolism and ATP production [14,15,16]. Recent studies have also highlighted the neuroprotective potential of tDCS in conditions like Parkinson’s disease [17] and stroke [18,19,20]. As a method of therapy, tDCS is gaining popularity among clinicians for its broad range of applications in psychiatric disorders, such as fibromyalgia [21], post-stroke recovery [18,19], depression [22], and schizophrenia [23,24]. In addition, clinical studies have shown specific effects of tDCS on memory, cognitive, and motor functions in both humans and rodents [5,13,24,25].

TMS employs rapidly changing magnetic fields to generate strong electrical fields in the brain, thus inducing action potentials in the neurons within the targeted area [26,27]. In the context of neuromodulation, TMS is most commonly applied in two main paradigms: repetitive transcranial stimulation (rTMS) and theta-burst stimulation (TBS). These paradigms typically use subthreshold magnetic pulses to modulate cortical excitability over time without inducing motor-evoked potentials. In rTMS, magnetic pulses are delivered at fixed frequencies. High-frequency rTMS (>5 Hz) has been shown to enhance cortical excitability, resembling the effect of anodal tDCS, whereas low-frequency rTMS (0–2 Hz) suppresses cortical activity, resembling the effect of cathodal tDCS, [28,29]. TBS, a more recent and efficient protocol, involves three short bursts of high-frequency stimulation (50 Hz) delivered at intervals of 200 ms (5 Hz) [30,31]. This protocol is subdivided into continuous TBS (cTBS) and intermittent TBS (iTBS). In cTBS, uninterrupted trains of three-burst pulses at 50 Hz are applied at 5 Hz for 20 s, resulting in prolonged cortical inhibition, resembling the effects of cathodal tDCS [30]. Conversely, iTBS consists of trains of three 50 Hz pulses delivered at 5 Hz for 10 s, repeated 20–30 times with 2 s intervals between trains, inducing sustained cortical excitation, analogous to anodal tDCS [30]. Notably, the neuromodulatory effects of TBS are comparable to or even exceed those observed with conventional rTMS, with the added advantage of reduced stimulation time [32,33]. This positions TBS as a powerful and practical technique. Placebo-controlled studies and clinical trials have demonstrated the efficacious potential of TMS in treating conditions like depression, migraines, pain, refractory epilepsy, tremors, dystonia, and compulsive disorders and as a neurorehabilitation method for patients with central nervous system disorders, such as trauma and stroke [28].

For many years, efforts have been made to understand the mechanism of action behind these techniques, and despite some gain of knowledge, the method of their employment in the clinical setup is still somewhat heuristic due to ongoing knowledge gaps. For example, it is still not well understood what is the cellular and molecular timeline of treatment effects and how long these effects linger. This impedes our ability to determine the optimal frequency of treatment that will enable the accumulation of beneficial effects and minimize adverse effects, which limits successful treatment. In addition, we still do not know enough to personalize treatments according to individual patients and their distinct disorders. Recent clinical trials have explored the value of combining neurostimulation techniques to enhance their individual effects [34,35] and augment pharmacotherapy [36,37]. However, limited understanding of their impact at the cellular and molecular levels, whether they are complementary or interfering, hinders the broad adoption of these paradigms.

This study reveals the long-lasting molecular changes induced by repetitive tDCS on cortical brain tissue. We compare our findings with previously reported gene expression changes immediately following single tDCS stimulation to assess the possible temporal dynamics of its effects on molecular mechanisms. Additionally, we examine the differential effects of tDCS and TBS on gene expression patterns to assess whether they cause distinct and/or overlapping gene expression changes in the long term. 

## 2. Results

### 2.1. Sustained Effect of Transcranial Direct Current Stimulation (tDCS) on Gene Expression

**Figure 1:**
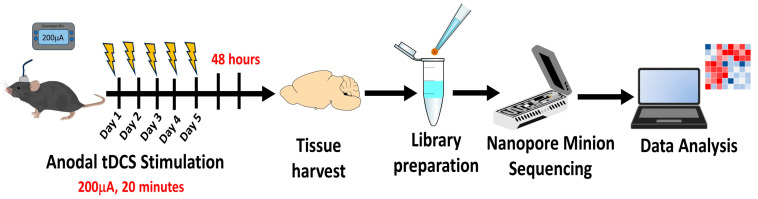
A schematic representation of the experimental protocol of tDCS followed by transcriptomic sequencing.

**Figure 2:**
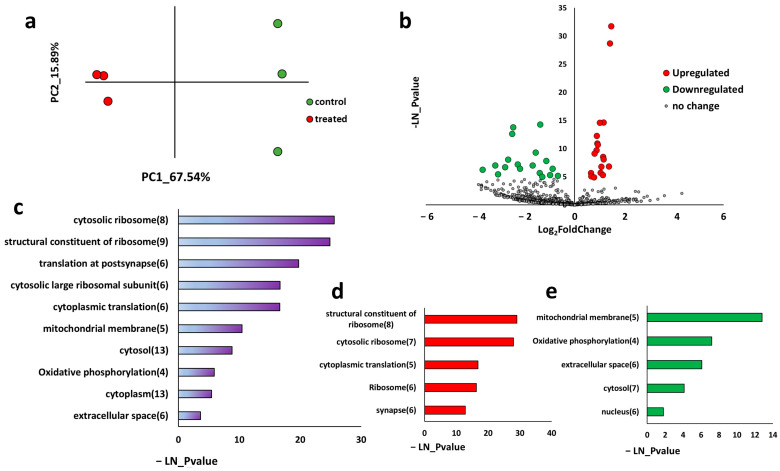
Transcriptomic analysis of the sustained effects of repetitive tDCS on gene expression 48 h post-stimulation. (**a**) Principal component analysis (PCA) of tDCS-treated and sham control samples. The x- and y-axes represent the first two principal components (PCs). Red dots indicate tDCS-treated samples, while green dots represent sham controls. (**b**) Volcano plot displaying log_2_ fold change (log_2_FC) on the *x*-axis and –ln(*p*-value) on the *y*-axis for all expressed genes. Significantly upregulated genes (*p*-value < 0.01, FC > 15%) are highlighted in red, and significantly downregulated genes (*p*-value < 0.01, FC < 15%) are highlighted in green. (**c**) Functional over-representation analysis of differentially expressed genes, showing the 10 most significantly enriched pathways. The *x*-axis represents –ln(*p*-value) for pathway enrichment, and the numbers in parentheses indicate the count of differentially expressed genes within each pathway. (**d**) Functional over-representation analysis of upregulated genes, highlighting the top five enriched pathways. (**e**) Functional over-representation analysis of downregulated genes, highlighting the top five enriched pathways.

To investigate the sustained effects of repetitive tDCS on gene expression, we generated an RNA sequencing library from the parietal cortices below the stimulation electrodes of tDCS-treated and sham-treated mice 48 h after the fifth and last treatment (Figure 1). Principal component analysis (PCA) [38] revealed distinct gene expression profiles between tDCS-treated and sham control mice (Figure 2a). Differential expression analysis identified 36 differentially expressed genes, with 18 upregulated and 18 downregulated genes (Figure 2b, Supplementary Table S1).

Functional over-representation of differentially expressed genes revealed that pathways related to translation and mitochondria, such as “cytosolic ribosome”, “translation at the post synapse”, “mitochondrial membrane”, and “oxidative phosphorylation” exhibited the highest enrichment (Figure 2c, Supplementary Table S2). When analyzed separately, upregulated genes were associated with pathways like “structural constituent of ribosome” and “cytosolic ribosome” indicating an increased effect on pathways related to protein translation as a sustained effect of repetitive tDCS (Figure 2d, Supplementary Table S3). On the other hand, downregulated genes were enriched in mitochondria-associated pathways, such as “mitochondrial membrane” and “oxidative phosphorylation” possibly indicating a mitochondrial response to tDCS in the long term (Figure 2e, Supplementary Table S4).

### 2.2. Immediate and Long-Term Effect of tDCS on Synaptic and Mitochondrial Gene Expression

To compare the long-term effects of tDCS 48 h after repetitive stimulation with its immediate effects on cortical gene expression, we utilized a previously published RNA-seq dataset that examined gene expression changes immediately following tDCS [39].

Differential expression analysis of the immediate effects of tDCS revealed 251 upregulated and 5 downregulated genes (*p* value < 0.01, FC ±15%) (Supplementary Figure S2, Supplementary Table S5).

**Figure 3:**
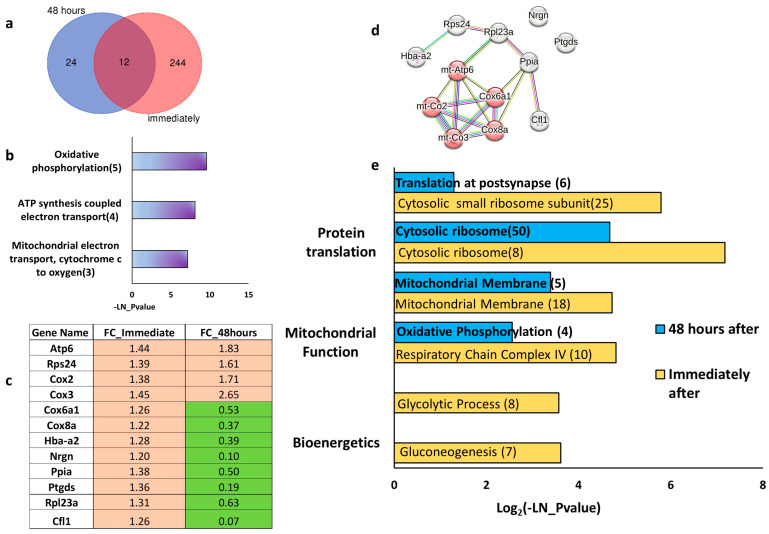
Comparison of gene expression immediately after a single tDCS session and 48 h post-repetitive tDCS stimulation. (**a**) Venn diagram illustrating differentially expressed genes shared between both time points. The blue circle represents 24 genes uniquely differentially expressed 48 h post-repetitive tDCS. The red circle contains 244 genes significantly differentially expressed only immediately after a single tDCS session. The overlap shows 12 genes differentially expressed at both time points. (**b**) Functional enrichment analysis of the 12 differentially expressed genes overlapping between the immediate single tDCS and the 48 h post-repetitive tDCS time points. The *x*-axis represents –ln(*p*-value) for pathway enrichment, and the numbers in parentheses indicate the count of differentially expressed genes within each pathway. (**c**) Table showing the fold change of the 12 overlapping differentially expressed genes across the two time points. Orange indicates an increase in fold change after tDCS compared to the sham control, while green represents a decrease. (**d**) STRING plot illustrating protein–protein interactions among the 12 overlapping differentially expressed genes at both time points. Connected nodes indicate known protein interactions, with red-highlighted nodes representing genes involved in oxidative phosphorylation. (**e**) Comparison of the long-term effect of repetitive tDCS with the immediate impact of tDCS on gene pathways. Functional enrichment analysis of all differentially expressed gene pathways categorized into protein translation, mitochondrial functions, and bioenergetics. The top two significantly enriched pathways for each category. Pathways differentially expressed 48 h post-repetitive tDCS are in blue, while those immediately after a single tDCS session are in yellow. The *x*-axis represents the logarithmic scale (log_2_) of-ln(*p*-value), and the numbers in parentheses indicate the count of differentially expressed genes associated with each pathway.

Comparison of datasets from immediate and long-term tDCS experiments found 12 common genes (Figure 3a). Functional enrichment analysis showed that these genes are enriched in mitochondria-associated pathways (Figure 3b). Notably, the expression patterns of these 12 genes differed significantly over time. Four genes, Atp6, Cox2, Cox3, and Rps24, remained upregulated both immediately and following repetitive tDCS. The other eight genes were upregulated immediately after a single tDCS session and downregulated 48 h after the last tDCS session (Figure 3c). Using STRING, we visualized the protein–protein interaction between these genes (Figure 3d), which showed that 10 out of the 12 genes are known to be physically interacting. Moreover, five of these genes are directly involved in oxidative phosphorylation.

When comparing the long-term effect of repetitive tDCS with the immediate impact of tDCS on the pathway level, we observed that in the short term, the most enriched pathways were associated with ribosomal protein translation (“cytosolic ribosome”, “cytosolic small ribosomal subunit”), mitochondrial function (“respiratory chain complex IV”, “mitochondrial membrane”), and bioenergetics (“gluconeogenesis”, “glycolytic processes”) (Figure 3e). However, 48 h after five days of repetitive stimulation, the enriched pathways were predominantly related to translation and mitochondrial function, with no enrichment of glycolysis-related pathways (Figure 3e, Supplementary Tables S2 and S6).

We predict that neurostimulation of any kind will ignite initial molecular effects that will further activate cascades of molecular pathways, thus producing a dynamic of sequential molecular effects over time. Although the initial pathways will decay, they will lead to long-term changes. One limitation of analyzing differentially expressed genes is its dependence on predefined statistical significance thresholds. Aiming to explore the dynamics of the aforementioned molecular pathways of protein translation and mitochondrial functioning, we performed Gene Set Enrichment Analysis (GSEA) for these specific pathways, a widely used approach for assessing pathway enrichment without restricting the analysis to differentially expressed genes [40,41].

We employed the Mouse Gene Informatics (MGI) collection of annotated pathways using protein-translation-related terms, such as “cytoplasmic translation”(GO:0002181) and “ribosome biogenesis” (GO:0042274, GO:0042273), for checking specific effects on genes involved in protein translation machinery, and “Mitochondrial Respiratory chain complex assembly”(GO:0033108) and “Cellular respiration” (GO:0045333) for checking the effects on genes related to specific mitochondrial functions and energy production.

**Figure 4:**
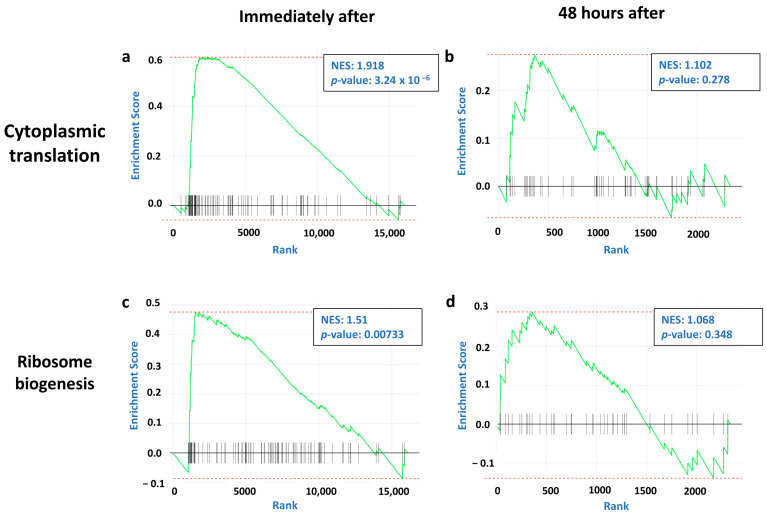
Immediate and sustained effects of tDCS on genes associated with protein translation show immediate activation of genes followed by decay over time. Gene Set Enrichment Analysis (GSEA) plots for two pathways regulating protein translation at two time points: immediately after a single tDCS session and 48 h post-repetitive tDCS. The *y*-axis represents the enrichment score (ES), and the *x*-axis denotes the ranking of genes by fold change. Vertical black lines represent the genes in each specific GO pathway. The green line indicates a running sum of the ES. Each panel shows the normalized enrichment score (NES) values and their corresponding *p*-value. (**a**,**b**) GSEA plots for the cytoplasmic translation pathway, analyzed (**a**) immediately after tDCS (NES: 1.918; *p*-value: 3.24 × 10^−6^) and (**b**) 48 h post-repetitive tDCS (NES: 1.102; *p*-value: 0.278). (**c**,**d**) GSEA plots for the ribosome biogenesis pathway, analyzed (**c**) immediately after tDCS (NES: 1.51; *p*-value: 0.00733) and (**d**) 48 h post-repetitive tDCS (NES: 1.068; *p*-value: 0.348).

We found that pathways regulating protein translation were upregulated immediately after single tDCS (Figure 4a,c; Supplementary Table S7). However, 48 h after repetitive tDCS (Figure 4b,d; Supplementary Table S8), these changes decayed and were no longer significant, suggesting that despite the broad upregulation of protein translation induced by tDCS immediately after stimulation, the effect decays in the long term.

**Figure 5:**
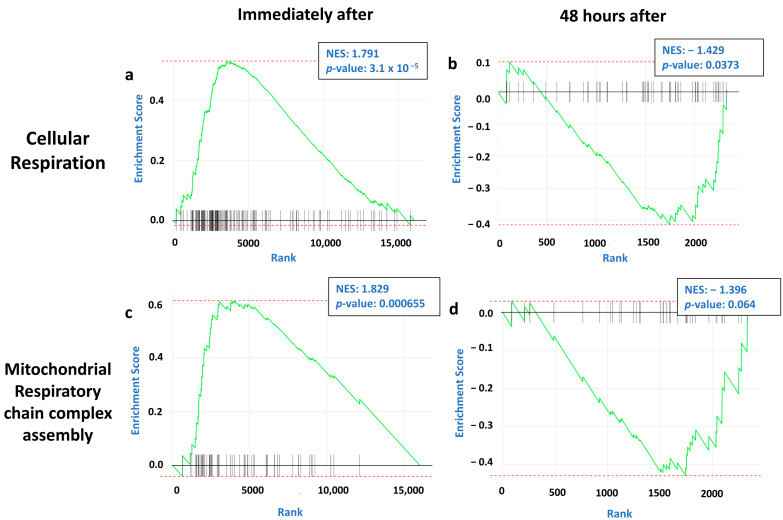
The immediate and sustained effects of tDCS on genes associated with mitochondrial functioning show a bimodal pattern of immediate gene activation followed by suppression and downregulation of genes over time. Gene Set Enrichment Analysis (GSEA) plots for two pathways regulating mitochondrial functioning at two time points: immediately after a single tDCS session and 48 h post-repetitive tDCS. The *y*-axis represents the enrichment score (ES), and the *x*-axis denotes the ranking of genes by fold change. Vertical black lines represent the genes in each specific GO pathway. The green line indicates a running sum of the ES. Each panel shows the normalized enrichment score (NES) values and their corresponding *p*-value. (**a**,**b**) GSEA plots for the cellular respiration pathway, analyzed (**a**) immediately after tDCS (NES: 1.791; *p*-value: 3.1 × 10^−5^) and (**b**) 48 h post-repetitive tDCS (NES: −1.429; *p*-value: 0.0373). (**c**,**d**) GSEA plots for the mitochondrial respiratory chain complex assembly pathway, analyzed (**c**) immediately after tDCS (NES: 1.829; *p*-value: 0.000655) and (**d**) 48 h post-repetitive tDCS (NES: −1.396; *p*-value: 0.064).

On the other hand, genes associated with mitochondrial function exhibited a bimodal temporal response to tDCS. Immediately after stimulation, mitochondrial pathways were significantly upregulated (Figure 5a,c; Supplementary Table S7). However, unlike protein translation, which simply decayed, mitochondrial-related pathways were actively downregulated 48 h after tDCS (Figure 5b,d; Supplementary Table S8), indicating that the initial enhancement of mitochondrial activity by tDCS is followed by a homeostatic downregulation, potentially preventing oxidative stress and preserving cellular stability. Overall, these results suggest that in the long term, tDCS regulates mitochondrial dynamics, promoting neuroprotection by decreasing mitochondrial activity and thus reducing the production of reactive oxygen species (ROS), which corresponds with previously published studies [42,43].

### 2.3. tDCS and TMS Elicit Distinct Gene Expression Changes in the Parietal Cortex

Recent clinical trials have shown that the beneficial effects of tDCS and TMS are enhanced when used together [34,44,45]. However, their distinct mechanisms, particularly at the genetic and molecular levels, remain unclear and largely not compared. To address this, we examined the long-term molecular effects of repetitive tDCS and repetitive TMS using the iTBS protocol. Specifically, we analyzed a recently published microarray dataset generated 48 h post-repetitive TMS from rat cortices [46].

Differential expression analysis of the iTBS dataset revealed 47 genes, with 25 upregulated and 22 downregulated (*p* value < 0.01, FC ±15%) (Supplementary Figure S3, Supplementary Table S9).

**Figure 6:**
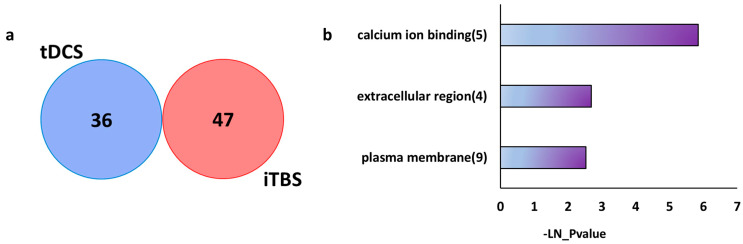
Comparison of sustained gene expression effects (after 48 h) of tDCS and iTBS. (**a**) Venn diagram shows no overlap of differentially expressed genes between the tDCS and iTBS datasets, 48 h post-stimulation. (**b**) Over-representation analysis of genes differentially expressed 48 h after iTBS, highlighting the top three most significantly enriched pathways. The *x*-axis represents –ln(*p*-value) for pathway enrichment, with the number of differentially expressed genes in each pathway shown in parentheses.

Comparison of differentially expressed genes 48 h after repetitive tDCS and 48 h after iTBS revealed no overlapping genes (Figure 6a). This suggests that these stimulation techniques induce distinct transcriptional changes in the brain. While tDCS induces changes in protein translation and mitochondrial functioning (Figure 2), iTBS differentially expressed genes were enriched in pathways like “calcium-ion binding”, “extracellular region”, and “plasma membrane” (Figure 6b, Supplementary Table S10).

This analysis suggests that in the long term, the two neuromodulatory techniques activate distinct molecular mechanisms, laying the groundwork for further exploration of their individual and combined therapeutic potential.

## 3. Discussion

Non-invasive brain stimulation has emerged as a powerful tool for modulating cortical plasticity; however, its long-term effects remain poorly understood. In this study, we systematically investigated the sustained effects of repetitive tDCS on cortical gene expression. Furthermore, we compared these long-term effects to the changes observed immediately after a single stimulation, providing a dynamic perspective on gene expression over time. In addition, we compared these sustained tDCS effects to the sustained effects induced by an alternative neuromodulatory technique, iTBS, a commonly used TMS protocol.

Our findings demonstrate that anodal repetitive tDCS induces long-lasting transcriptional changes in the parietal cortex. These changes are characterized by the upregulation of protein-translation-related genes, alongside the downregulation of genes involved in mitochondrial function (Figure 2). These results align with prior studies suggesting that tDCS exerts long-term plasticity effects and modulates metabolic pathways [10,11,16,47]. The observed increase in translation-related genes suggests that tDCS promotes protein synthesis, a mechanism critical for experience-dependent, long-term plasticity. This is consistent with previous studies that demonstrate that tDCS induces the enhancement of protein-translation-related genes, promoting synaptic plasticity [48,49]. Meanwhile, the reduction in mitochondrial gene expression may reflect an adaptive shift in cellular energy demand, potentially linked to its activity-dependent homeostasis [50,51].

By comparing long-term effects of repetitive tDCS to the immediate changes after single stimulation (Figure 3), we aimed to distinguish transient transcriptional responses from sustained adaptations, offering deeper insight into the temporal dynamics of cortical response to tDCS. As observed, the immediate transcriptional response to tDCS was marked by significant upregulation of genes related to translation, mitochondria, and glycolytic pathways, indicating an immediate surge in bioenergetic mechanisms. Forty-eight hours after the last repetitive stimulation, the expression of the genes related to translation machinery was no longer enhanced, while the expression of mitochondria-related genes was even suppressed (Figure 4 and Figure 5). This indicates that tDCS initially activates protein synthesis, required for producing long-term changes, alongside igniting mitochondrial and glycolytic bioenergetic machinery to provide the required energy for such processes. It is suggested that over time, once the required proteins are synthesized, the translation machinery slows down to prevent proteostasis. At the same time, to protect the neurons from excessive oxidative stress and cellular damage associated with prolonged energy overproduction, the bioenergetic machinery is downregulated in two separate pathways, each with a different dynamic. The glycolytic machinery, which is an immediate cellular response to elevated energy requirements, is turned down to reach baseline levels in the long term, whereas the mitochondrial machinery, which is a more sustained form of energy production that entails the hazard of oxidative stress production, is actively suppressed.

Overall, the temporal divergence between protein-translation-related genes and mitochondrial function highlights the dynamic nature of tDCS effects and lays the groundwork for understanding how these adaptations unfold over time. Although we are comparing datasets from different species, mice in the long-term experiment and rats in the single short-term stimulation experiment, this cross-species perspective still provides valuable insight into the temporal dynamics of tDCS-induced molecular changes, despite its limitations. Importantly, the stimulation parameters used in the rat dataset and in our study are aligned with those commonly employed in human protocols [52], thereby supporting the translational relevance of our findings.

In addition, our findings highlight key distinctions in the sustained transcriptional effects of tDCS and iTBS, reinforcing the notion that these neuromodulatory techniques engage different molecular pathways over time. Recently conducted clinical trials have highlighted how usage of the two techniques together boosts their individual effects [44,45]. We observed that while tDCS primarily influences mitochondrial function and translation, iTBS exerts its effects on calcium-related processes, with no overlap in their long-term gene expression profiles. This divergence suggests that the therapeutic benefits of combining these techniques may arise from complementary mechanisms.

## 4. Materials and Methods

### 4.1. Animals

Six 8-week-old male C57BL/6 mice were used for the experiments. All experimental protocols followed the US National Institutes of Health guidelines and were approved by the Institutional Animal Care and Use Committee of the University of Haifa under ethics number 1334 U. The mice were maintained throughout the experiment in a 12 h light/dark cycle with 22 ± 1 °C ambient temperature, with food and water ad libitum. Experiments were consistently conducted during the light phase of the cycle.

### 4.2. Surgical Procedure

The mice were anesthetized with an intraperitoneal injection containing fentanyl 0.05 mg/kg, midazolam 5 mg/kg, and medetomidine 0.5 mg/kg before undergoing a surgical procedure to adhere the stimulation electrodes on the revealed skull. A warming blanket was used to maintain body temperature, and the eyes of the mice were kept moist using 5% synthomycine ointment (containing 5% chloramphenicol) throughout the procedure. After sedation, the fur on the mice’s heads was shaved, and an incision was made between the bregma and the lambda. The exposed tissue was cleaned sequentially with 0.9% saline, diluted hydrogen peroxide, and 70% ethanol.

A custom-made plastic cylinder (4 mm external diameter) was used to mark the skull at the sagittal suture and the lambda junction. The cylinder was secured with cyanoacrylate glue (Surgibond, SMI, St. Paul, MN, USA) at its circumference, and, around the cylinder, we applied a two-component dental cement (Unifast Trad, GC Corporation, Tokyo, Japan). Once the cement partially hardened, the cylinder was removed, leaving a 4 mm cavity exposing the skull at its base. That cavity was filled with a conducting silver-based epoxy glue (Silver Conduc Epoxy 21G, MG Chemicals, Burlington, Ontario, Canada), and a multithread copper wire was inserted into the conducting glue to adhere to it, ensuring stable and continuous connectivity between the copper wire and the skull during stimulation (Supplementary Figure S1). After the procedure, the mice received normal glucose, analgesic medication (Meloxicam 5 mg/kg), antibiotics (Baytril 7.5 mg/kg), and an anti-sedative mix (Naloxone 1.2 mg/kg, Flumazenil 0.5 mg/kg, Atipamezole 2.5 mg/kg) before being placed in a warm cage for recovery.

### 4.3. tDCS Treatment

Following the surgical procedure, mice were allowed to recover for five days before being stimulated either by anodal tDCS using the Neuromyst Pro device or sham stimulation. Mice were randomly assigned to either the tDCS treatment group or the sham group.

Before each session, mice (treated and sham) were lightly and briefly sedated with isoflurane to facilitate fitting a custom-made jacket containing the cathode electrode with a diameter of 3 cm (CF3200 ValuTrode, Axelgaard Manufacturing Co., Ltd., Fallbrook, CA, USA) and conductive gel (Spectra 360, Parker Laboratories, Inc., Fairfield, NJ, USA). Stimulation for each animal was initiated only after they regained full consciousness and motion. To ensure consistency and minimize stress, the stimulation sessions included a 30 s ramp-up and ramp-down phase to provide gradual transitions in current intensity. In addition, we confirmed that the resistance remained less than 20 kΩ, ensuring that stimulation was continuous and stable throughout. tDCS-treated mice received 20 min 0.2 mA (current density of 1.59 mA/cm^2^) daily sessions over five consecutive days. Sham-stimulated mice underwent all procedures as the treated mice but received only a 30 s ramp-up and ramp-down phase to induce sensory stimulation without the actual 20 min tDCS stimulation. During these sessions, the mice remained awake and freely mobile.

### 4.4. Tissue Harvesting

Forty-eight hours after the fifth and final tDCS stimulation, mice were euthanized through cervical dislocation, brains were quickly extracted in ice-cold, phosphate-buffered saline (PBS), and the parietal cortex below the stimulating electrode was isolated and snap-frozen in liquid nitrogen and stored at −80 °C until further analysis. 

### 4.5. Sequencing

To isolate RNA, tissue was homogenized in TRI-based reagent on ice and processed further according to the instructions on the Zymo Direct zol RNA Miniprep kit (Zymo Research, Irvine, CA, USA; Cat# R2051A). Phase separation was used for isolating pure RNA samples, which were then eluted in DNAse/RNAse-free water. It was later quantified for purity and concentration using a NanoDrop spectrophotometer. The prepared samples were then stored at −80 °C for further analysis.

In accordance with the instructions of Oxford nanopore “Rapid Sequencing DNA V14—barcoding protocol” [53], 400 μg of isolated total RNA per sample was reverse-transcribed with cDNA-RT adaptor and Annealing buffer at 60 °C for 5 min, followed by ligation with NEBNext^®^ Buffer (NEB, Ipswich, MA, USA; Cat#B6058) and T4 DNA Ligase (NEB, Ipswich, MA, USA; Cat# M0202M). After exonuclease treatment, samples were purified using RNAClean XP beads (Beckman Coulter^TM^ Life Sciences, IN, USA; Cat#A63987). Strand-switching was initiated with RT-Primer, dNTPs, and Strand-switching primer II (SQK-RBK114.24). This was followed by the addition of Maxima H minus Reverse Transcriptase (ThermoFisher, Waltham, MA, USA, Cat#EP0751) and incubation of the reaction in a thermal cycler using defined thermal cycling conditions. Next, reverse-transcribed cDNA (5 ml) was amplified using LongAmp Hot Start Taq (NEB, Ipswich, MA, USA; Cat#M0533S) with barcode primers (SQK-RBK114.24) under optimal cycling conditions. Unbound primers were degraded with Exonuclease I (NEB, Ipswich, MA, USA; Cat#M0293S) for 20 min. Samples were purified using AMPure XP beads (Beckman Coulter^TM^ Life Sciences, IN, USA; Cat # A63881), washed with 70% ethanol, and resuspended in Elution Buffer for downstream analysis. Equimolar cDNA quantities were then taken per sample, pooled into a library, and loaded into the flow cell FAZ07279 in the Minion Mk1b device (Oxford Nanopore Technologies, Oxford, UK) according to the instructions from the kit SQK-RBK114.24. Base calling and demultiplexing of reads were performed with Oxford nanopore built-in software MinKNOW (Min24.06.5).

Raw sequencing reads that passed quality control (Qscore > 6) were aligned on the reference genome (GRCm38) [54] using the minimap2 alignment tool (Version 2.28, https://github.com/lh3/minimap2) [55]. Gene expression quantification was performed using NanoCount (Version 1.1.0, https://a-slide.github.io/NanoCount/nanocount_io/) [56], and normalization and differential expression analysis were conducted using the DeSeq2 package (Bioconductor version 3.21) [57]. Thresholds for differentially expressed genes were set to *p*-value < 0.01 and a fold change of ±15%.

### 4.6. Publicly Available Datasets

To compare the sustained molecular effects of tDCS with its short-term effects, we utilized a previously published RNA-seq dataset collected immediately after tDCS and sham stimulation from the cerebral cortices of wild-type male Sprague Dawley rats [39]. The experimental tDCS protocol utilized stimulation parameters consistent with those employed in our study, specifically cortical stimulation using anodal tDCS at 250 µA for 20 min. Raw demultiplexed FASTQ files were directly obtained from the authors [39]. As we previously described [16], the raw files were aligned to the rat genome (mRatBN7.2, INSDC Assembly) using a splice-aware HiSat2 algorithm [58]. Gene expression profiles were calculated using the RSEM algorithm [59]. Gene expression normalization and differential gene expression analysis were performed utilizing DeSeq2 [57]. Thresholds for differentially expressed genes were set to *p*-value < 0.01 and a fold change of ±15%.

For comparison of tDCS and TMS effects, we utilized recently published microarray gene expression data [46]. The z-score log-normalized gene expression table generated by the authors of the study was downloaded directly from NCBI Gene Expression Omnibus (GSE230149). Similarly to our experimental approach, we utilized the gene expression data from in vivo experiments generated from parietal cortices of young (6–7 months) wild-type Long-Evans rats treated with either iTBS or sham. Differential expression analysis was performed using ANOVA. Thresholds for differentially expressed genes were set to *p*-value < 0.01 and a fold change of ±15%.

### 4.7. Functional Analysis of Gene Expression

Over-representation analysis (ORA) was performed using DAVID (Knowledge version v2024q4, https://davidbioinformatics.nih.gov/) [60]. Gene Set Enrichment Analysis (GSEA) [40,41] was performed using ClusterProfiler (Version 4.12.2) [61], and visualizations were generated using the ggplot2 R package (Version 3.5.1) [62].

## 5. Conclusions

In conclusion, our study demonstrates that tDCS induces distinct, time-dependent gene expression changes in the cortex, with both short and long-lasting effects on translation pathways, a transient short-term impact on glycolysis, and a bimodal effect on mitochondrial-related genes of upregulation in the short term, followed by downregulation in the long term. Comparison of the transcriptomic changes of tDCS and TMS over time shows that these two therapies activate different molecular pathways. Overall, our findings provide new insights into the neurobiological mechanisms underlying tDCS, highlighting its potential for therapeutic applications and its differentiation from TMS. The observed transcriptional shifts reinforce the idea that tDCS supports both immediate and sustained adaptations in the cortical circuits, paving the way for future research on its synergistic use with other neuromodulatory techniques.

## 6. Limitations of the Study

This study utilized three distinct datasets from different animal models using slightly different stimulation protocols and analyzed using three sequencing methods: Illumina, nanopore, and microarray. These variations pose challenges in ensuring consistent comparisons. However, the use of independent datasets enhances the robustness of our findings, as converging results across different models and methodologies suggest truly global effects. The use of male mice exclusively also represents a limitation, as the results may not be fully generalizable to females. In addition, our study lacks more time points to determine a time course that will exhibit the dynamics in detail. Moreover, this was a transcriptomic study, and while the results reflect changes at the mRNA level, they may not directly translate to functional protein alterations. Further studies may be warranted to validate these findings. Despite these challenges, this study provides the first comparative transcriptional analyses of short- and long-term effects of tDCS as well as a comparison to long-term TMS, offering insights into their distinct and overlapping molecular mechanisms and laying the groundwork for future research.

## Data Availability

The data generated for this article have been deposited in the NCBI database under the accession ID PRJNA1231775. Details about the other datasets used in this study are contained within the article and provided in the supplementary information.

## Supplementary Materials

The following supporting information can be downloaded at https://www.mdpi.com/article/10.3390/ijms26178634/s1.

## Abbreviations

The following abbreviations are used in this manuscript:

tDCS	Transcranial Direct Current Stimulation
TMS	Transcranial Magnetic Stimulation
iTBS	Intermittent Theta Burst Stimulation
PCA	Principal component analysis
GSEA	Gene Set Enrichment Analysis
