# Supplementary figures and images for: From Immediate Impact to Enduring Change: A Transcriptomic Comparison of tDCS’s Temporal Effects and Its Long-Term Equivalence with TMS
Source: Int J Mol Sci. 2025 Sep 4;26(17):8634. doi: 10.3390/ijms26178634 (PMC12429358; doi:10.3390/ijms26178634)

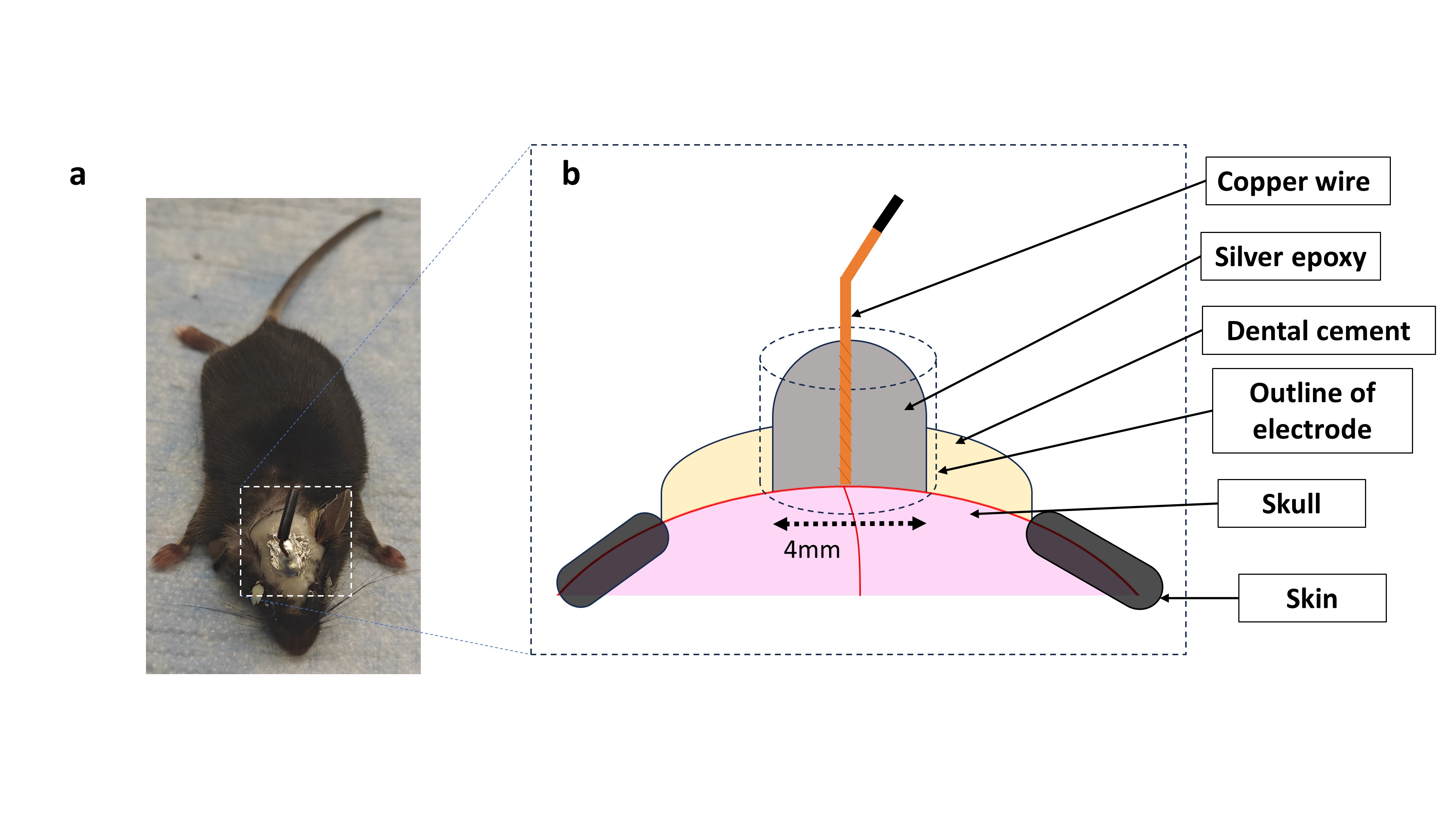

Supplement: Supplementary file 1 [file ijms-26-08634-s001.zip › Supplementary Figure S1.jpeg]

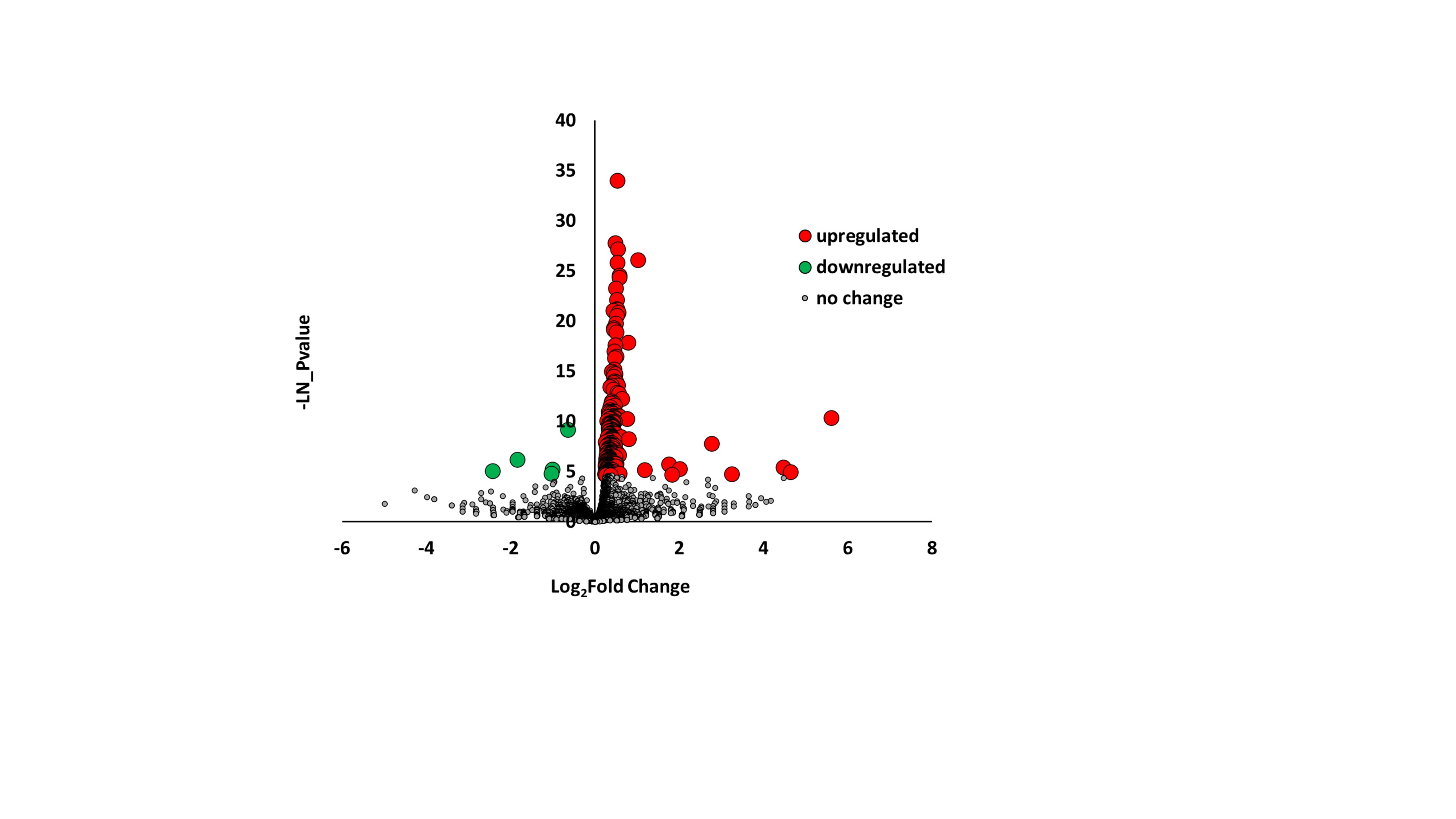

Supplement: Supplementary file 1 [file ijms-26-08634-s001.zip › Supplementary Figure S2.jpeg]

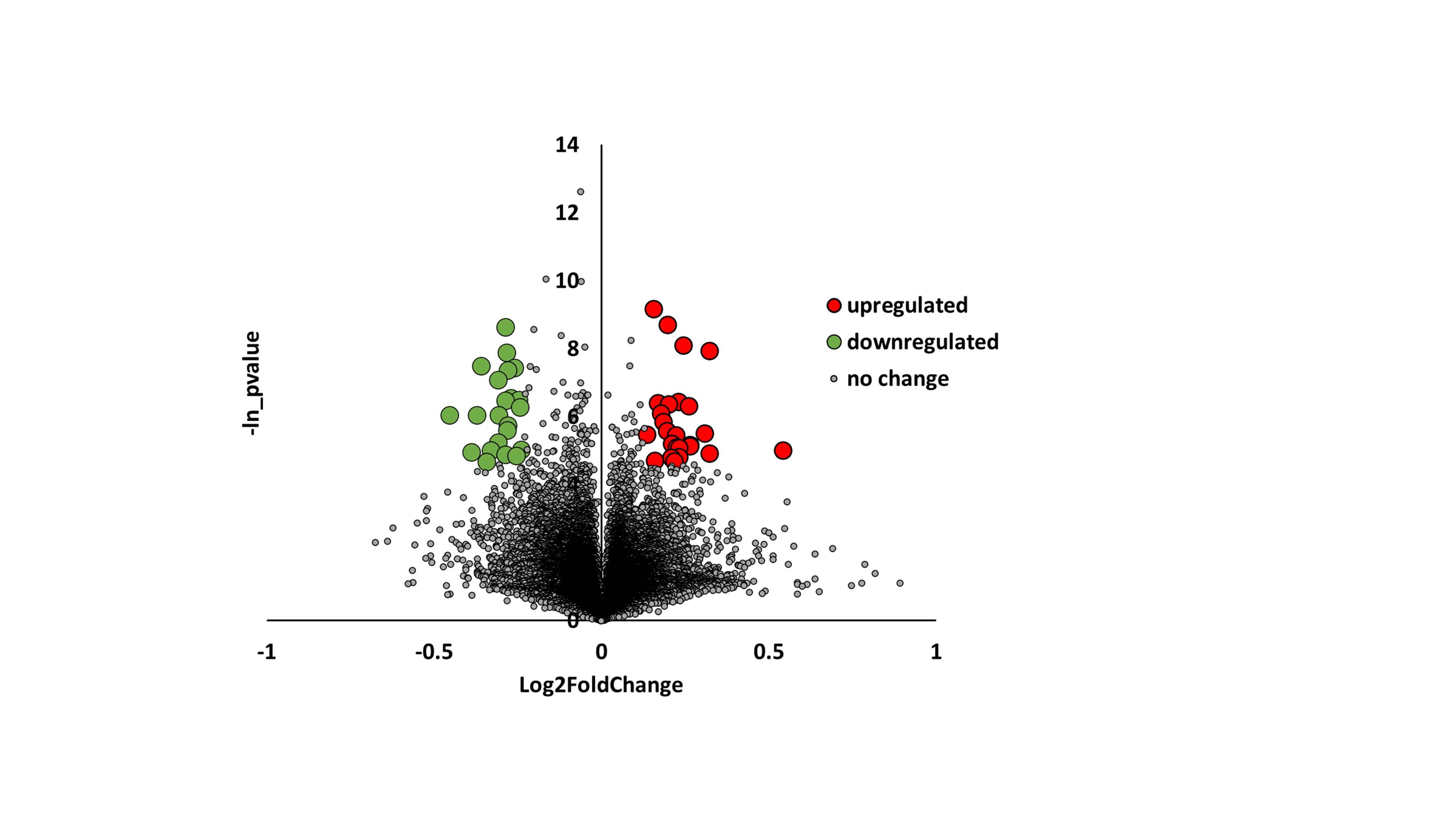

Supplement: Supplementary file 1 [file ijms-26-08634-s001.zip › Supplementary Figure S3.jpeg]
